# Eigen-entropy based time series signatures to support multivariate time series classification

**DOI:** 10.1038/s41598-024-66953-7

**Published:** 2024-07-12

**Authors:** Abhidnya Patharkar, Jiajing Huang, Teresa Wu, Erica Forzani, Leslie Thomas, Marylaura Lind, Naomi Gades

**Affiliations:** 1 School of Computing and Augmented Intelligence, Arizona State University, Tempe, AZ 85281 USA; 2 ASU-Mayo Center for Innovative Imaging, Arizona State University, Tempe, AZ 85281 USA; 3 The Biodesign Institute, Arizona State University, Tempe, AZ 85287 USA; 4 Division of Nephrology and Hypertension, Department of Internal Medicine, Mayo Clinic in Arizona, Scottsdale, AZ USA; 5 Department of Comparative Medicine, Mayo Clinic in Arizona, Scottsdale, AZ USA

**Keywords:** Correlation coefficient, Eigenvalue, Multivariate time series classification, Dense multi scale entropy, Eigen-entropy, Time series signatures, Health care, Applied mathematics, Computational science, Information technology, Software, Statistics

## Abstract

Most current algorithms for multivariate time series classification tend to overlook the correlations between time series of different variables. In this research, we propose a framework that leverages Eigen-entropy along with a cumulative moving window to derive time series signatures to support the classification task. These signatures are enumerations of correlations among different time series considering the temporal nature of the dataset. To manage dataset’s dynamic nature, we employ preprocessing with dense multi scale entropy. Consequently, the proposed framework, Eigen-entropy-based Time Series Signatures, captures correlations among multivariate time series without losing its temporal and dynamic aspects. The efficacy of our algorithm is assessed using six binary datasets sourced from the University of East Anglia, in addition to a publicly available gait dataset and an institutional sepsis dataset from the Mayo Clinic. We use recall as the evaluation metric to compare our approach against baseline algorithms, including dependent dynamic time warping with 1 nearest neighbor and multivariate multi-scale permutation entropy. Our method demonstrates superior performance in terms of recall for seven out of the eight datasets.

## Introduction

Time series classification is a type of supervised machine learning classification problem where the instances in time series datasets are ordered. The temporal aspect of the dataset adds a layer of complexity to the time series classification problems. If any of the machine learning methods e.g., support vector machine (SVM) or logistic regression (LR) etc. is used to classify raw time series datasets, it is possible that some important temporal characteristics are missed by the classification algorithm. Also, in the past, extensive research has focused on univariate time series, whereas, in real world scenarios, we encounter cases of multivariate time series more often. For multivariate time series, inter-dimensional dependency among different time series might be as important as an autocorrelation in an individual time series. It is thus important that multivariate time series algorithm is designed to take into consideration an autocorrelation in the time series along with the correlation among time series of various dimensions. Traditionally, time series classification can be categorized into distance-based approaches, feature-based approaches, ensemble-based approaches, and recently developed deep learning (DL) approaches^1,2^.

Distance-based approaches use similarity measures like Euclidean distance or Dynamic Time-Warping (DTW) for classification. K-Nearest Neighbors (KNN) with DTW is a standard for time series classification^3^. Typically, one-nearest neighbor with DTW is used for univariate time series classification. For multivariate time series, DTW has two methods: independent warping (DTWI) and dependent warping (DTWD). DTWI treats each dimension independently and sums the DTW distances, while DTWD assumes the same warping across all dimensions. M. Shokoohi-Yekta proposes adaptive warping (DTWA), choosing between DTWI and DTWD based on a threshold from training data^4^.

Feature-based methods extract features that capture the global and local patterns of time series. These features are used to create a Bag-of-Words^5^, which is then input to classifiers. Various features can serve as inputs for feature-based methods; for instance, the bag-of-features framework^6^ uses subsequences, while Bag-of-Symbolic Fourier Approximations (SFA)-Symbols (BOSS)^7^ employs substructures.

In case of model ensemble approaches, a collection of classifiers is used. Elastic ensemble^8^ combines 11 different nearest neighbor (NN) classifiers based on elastic distance measure. The collective of transformation ensembles (COTE)^9^ is a combination of classifiers in the time, autocorrelation, power spectrum and shapelet domains. Canonical Interval Forest (CIF) classifier^10^ combines time series forest^11^ with catch22^12^, a feature set with 22 time series features that provide diverse and descriptive characteristics.

Recently, deep learning (DL) models have become popular for classifying biomedical temporal data^13^. DL employs neural networks composed of multiple layers of neurons. Residual Network (ResNET)^14^ is a DL algorithm that has been effectively used for time series classification^2^. The connections within ResNET enhance gradient flow, mitigating vanishing gradient problems. Inception Time is another DL algorithm that adapts ResNET by incorporating Inception modules^15^. H. Ismail Fawaz introduced Inception Time, which consists of an ensemble of 5 inception networks^16^. RandOM Convolutional KErnal Transform (ROCKET)^17^ transforms the time series with random convolutional kernels. The transformed features with these kernels are then used for classification with linear classifiers (ridge or logistic regression). DL algorithms typically need a large dataset for training. Additionally, DL algorithms are often not considered highly interpretable.

In addition to above-mentioned algorithms, different types of entropy measures have been proposed in the literature for time series classification. Entropy, as it relates to dynamical systems, is the rate of information production. Entropy measures different properties of the time series namely complexity, predictability, uncertainty, just to name a few. For example, permutation entropy (PE)^18^ measures the complexity using ordered subsequences of the time series to assess temporal structure of a sequence to perform classification. Another popular approach using entropy is based on approximate entropy (ApEn)^19^ that measures amount of regularity in the time series. Sample entropy (SampEn) improves upon ApEn^20^. Dispersion entropy (DE)^21^ quantifies time series regularity, addressing limitations of previous measures like PE and SampEn. Fuzzy Entropy (FuzzyEn)^22^, based on fuzzy sets, enhances SampEn by measuring the similarity of vector shapes.

Entropy increases with disorder and is highest for random systems. However, higher entropy does not necessarily indicate greater dynamical complexity. Many dynamic systems operate over multiple temporal or spatial scales, leading to incomplete descriptions of their dynamics. For example, diseased subjects often show reduced entropy compared to free-running healthy subjects^23^. Costa introduced multi-scale entropy (MSE) to measure biological system complexity using heart rate time series^24^. However, MSE requires scale factors to be limited due to sensitivity to series length, and traditional MSE methods use positive integers with fixed step sizes, which can limit analysis precision. To address these limitations, a new method called dense MSE (DMSE) is proposed^25^. DMSE expands the original sequence while preserving its characteristics, then refines it using more precise scale factors for better results.

The proposed time series classification methods (excluding entropy-based methods) have been adapted from univariate to multivariate applications by either forming an ensemble of models built independently on each dimension or by randomly selecting dimensions to transform the time series for classification. Entropy-based methods effectively classify univariate time series, but their application to multivariate time series, though proposed^26,27^, remains unsatisfactorily developed.

As previously discussed, it is crucial to account for the interdependencies among time series across various dimensions when conducting multivariate time series classification. Our primary focus is on detecting diseases, which involves a binary classification task. The relationships between different dimensions of biomedical temporal data, frequently utilized for disease detection, are well-established^28^. Additionally, clinical trials often suffer from limited data^29,30^. Therefore, our classifier must handle limited data while achieving high recall, as misclassifying diseased subjects as healthy is undesirable^31,32^. While many accurate classifiers e.g. ROCKET^17^, Hierarchical Vote Collective of Transformation-based Ensembles (HIVE-COTE)^33^ as mentioned by AP Ruiz^3^ exist, they extract complex features from various time series transformations. However, their lack of explainability^34^ makes them difficult for clinicians to understand. This is a common issue for researchers collaborating with medical professionals. Consequently, our goal is to develop novel temporal features for a classification algorithm that clinicians can easily comprehend. We thus introduce a new framework that presents an innovative method for classifying multivariate time series data by emphasizing the correlations among various dimensions. This framework aims to achieve high recall rates even with small clinical data sets, ensuring that the algorithm is explainable to clinicians.

Prior research suggests that changes in parameter values occur before the onset of disease, making them valuable indicators for early detection. For example, in sepsis detection, previous studies have identified heart rate (HR), diastolic blood pressure (DBP), systolic blood pressure (SBP), mean arterial pressure (MAP), and respiratory rate (Resp) as critical indicators^35^. These parameters begin to change before the onset of septic shock and exhibit different correlations among each other for the person who is likely to get a septic shock compared to the healthy person. By considering the temporal aspect of the multivariate time series data, our framework aims to identify these correlations and detect the presence of disease. To capture this correlation among different features of the multivariate data, we use Eigen-entropy (EE) technique developed by J. Huang^36^. It should be highlighted that this is the inaugural application of EE in the classification of multivariate time series.

EE is a measure of feature heterogeneity in a dataset. It is useful for identifying correlations between features, with low values indicating strong correlation and high values indicating heterogeneity. This property is particularly relevant for assessing the degree of asynchronization among multiple features, which is expected to differ between healthy individuals and those with a disease. However, EE is not suited for temporal data, as it cannot capture information of the past data points. To address this limitation, we propose using a cumulative moving window (CMW). This approach involves gradually including more data points from a multivariate time series until the entire length of the time series is covered. By including past data points and generating features that reflect changes in EE over different CMWs, we can account for the trends of the time series. In other words, CMW helps us memorize the information of the past data points. The features, termed as time series signatures in this context, serve to describe a specific multivariate time series for a given CMW. It is crucial to highlight that our framework ensures complete preservation of information, as each dimension plays a role in computing the EE value within a unified CMW. Moreover, the use of CMW ensures that our method retains the temporal properties of the time series by keeping the information from earlier data points.

Multivariate time series datasets are highly dynamic and complex, requiring multiple temporal scales to capture their information adequately. To address this, we transform the original time series into multiple other time series with different temporal scales, using dense multi scale entropy^25^. For each transformed dataset, we repeat the process described above, derive the Eigen-entropy based Time Series Signatures ($EE-TSS$) which can be used for multivariate time series classification task.

To summarize, we present some key contributions to the field of multivariate time series classification for disease detection. Firstly, we introduce a novel framework that emphasizes the correlations among various dimensions of biomedical temporal data, leveraging the EE technique for the first time in this context. Our approach incorporates a CMW to extend EE’s applicability to temporal data, capturing trends and preserving past information. This innovative method not only achieves high recall rates with limited clinical datasets but also ensures the algorithm’s feature generation is understandable, addressing a critical need for clinician-friendly tools. Furthermore, we develop new temporal features termed time series signatures, which suffer no loss of information due to consideration of all the dimensions. Our contributions thus demonstrate the potential for multivariate time series classifiers that can be used in clinical settings for disease detection.

The rest of the paper is organized as follows. The design of $EE-TSS$ and details of preprocessing of the datasets are presented in the “Methods” section. The results obtained from the application of $EE-TSS$ on UEA datasets^37^, gait dataset^38^, and an institutional sepsis dataset from the Mayo clinic are presented in the “Experiments and results” section. The “Discussion” section discusses the advantages and limitations of $EE-TSS$ approach. Finally, the conclusions are drawn, and stated in the “Conclusion” section along with the required future work.

## Methods

In this section, we present a brief description of dense multi scale entropy employed for data preprocessing, the Eigen-entropy, and explain how $EE-TSS$ is developed using Eigen-entropy in conjunction with cumulative moving window for multivariate time series classification.

### Dense multi scale entropy for data preparation

Many dynamic systems operate over multiple temporal scales. It is thus possible to have inaccurate or incomplete descriptions of the underlying dynamics in these dynamic systems. M. Costa introduces the multi-scale entropy (MSE) analysis and applies it to measure the complexity of the time series of heart rates^24^. However, since the MSE allows use of only integer scale factors, it makes its application challenging for shorter time series since the scale factor indicates the number of corresponding time points averaged to calculate the entropy value. Dense multi scale entropy (DMSE) is thus introduced by Zhao^25^ to enable the use of non-integer scale factors by expanding the time series. The first step of calculating DMSE is performing a coarse-graining procedure on the original time series. The coarse-graining procedure has two parts to it. If the raw time series is given by ${t(i),i=1,2, \ldots ,N}$, and the scale factors are integer values, DMSE follows Eq. (1). If the scale factors are non-integer values, DMSE follows Eq. (2). Here, $\tau$ is the non-integer scale factor, and $\alpha$ represents the minimum step size of the scale factor, $Q = \tau /\alpha$, $s = (1/\alpha ) * N$.

1 $$\begin{aligned} \begin{gathered} \textrm{y}_{\textrm{j}}^{\tau (\alpha )}=\frac{1}{\tau } \sum _{i=(j-1) \tau +1}^{j \tau } t(i), j=1,2, \ldots , \frac{N}{\tau }; \end{gathered} \end{aligned}$$

2 $$\begin{aligned} \textrm{y}_{\textrm{j}}^{\tau (\alpha )}=\frac{1}{Q} \sum _{s=(j-1) Q+1}^{j Q} t(s). \end{aligned}$$

Step size is suggested to be a decimal divisible by 1. The up-sampled series is obtained from the raw time series by inserting ($\frac{1}{\alpha }$ – 1) zero value points between adjacent data points followed by filtering. If the time series, represented by *z*(*s*), is obtained by adding zero points between adjacent data points, the filtered time series *t*(*s*) is derived from *z*(*s*) by applying a fourth-order low-pass Butterworth filter, following the guidelines of the paper by Zhao^25^. Time series *z*(*s*) is obtained using a following Eq. (3). *t*(*s*) is obtained by filtering *z*(*s*).

3 $$\begin{aligned}{} & {} z(s)=\left\{ \begin{array}{c} t(s), s=1; \\ t\left( \frac{s-1}{1 / \alpha }+1\right) , s=1 / \alpha +1,2 / \alpha +1,...,(N-1) / \alpha +1;\\ 0, \text{ other }. \end{array}\right. \end{aligned}$$

4 $$\begin{aligned}{} & {} t(s)= \text{ filter } (z(s)). \end{aligned}$$

**Figure 1:**
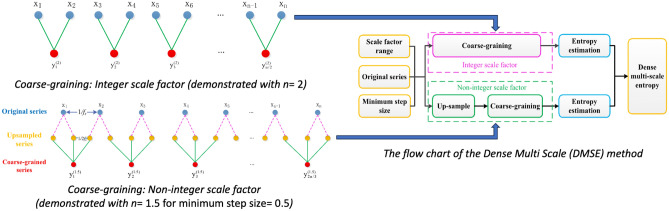
The flow chart of the dense multi scale entropy method^25^.

Once the upscaled time series is obtained, *Q* is adopted to determine the data segment length for the coarse-graining procedure under non-integer scale factors and obtain the coarse-grained sequence according to Eq. (2). Zhao proposes use of SampEn over the coarse-grained time series to form DMSE matrix^25^. The flowchart for calculating DMSE,  derived from the cited paper, is presented in Fig. 1 below.

### Eigen-entropy

Let ${\textbf{X}} \in R^{n \times m}$ denote a time series dataset that contains *m* time series corresponding to its *m* different feaures, each having *n* values or time points collected over time interval. We can represent ${\textbf{X}}$ as a matrix:

5 $$\begin{aligned} {\textbf{X}}=\left[ {\textbf{x}}_1, \ldots , {\textbf{x}}_{{\textbf{n}}}\right] ^{{\textbf{T}}}=\left( \begin{array}{cccc} x_{11} &{} x_{12} &{} \cdots &{} x_{1 m} \\ x_{21} &{} x_{22} &{} \cdots &{} x_{2 m} \\ \vdots &{} \vdots &{} \ddots &{} \vdots \\ x_{n 1} &{} x_{n 2} &{} \cdots &{} x_{n m} \end{array}\right) , \end{aligned}$$

where ${\textbf{x}}_i=\left[ x_{i 1}, \ldots , x_{i m}\right] , i=1, \ldots , n$. The correlation coefficient matrix on the feature space of ${\textbf{X}}$ is defined as,

6 $$\begin{aligned} {\textbf{C}}=\frac{1}{n} {\textbf{X}}_{{\textbf{S}}}^{{\varvec{T}}} {\textbf{X}}_{{\textbf{S}}}=\left( \begin{array}{cccc} 1 &{} c_{12} &{} \cdots &{} c_{1 m} \\ c_{21} &{} 1 &{} \cdots &{} c_{2 m} \\ \vdots &{} \vdots &{} \ddots &{} \vdots \\ c_{m 1} &{} c_{m 2} &{} \cdots &{} 1 \end{array}\right) , \end{aligned}$$

where,

7 $$\begin{aligned} {\textbf{X}}_{{\textbf{S}}}=\left( \begin{array}{cccc} \frac{x_{11}-\mu _1}{\sigma _1} &{} \frac{x_{12}-\mu _2}{\sigma _2} &{} \cdots &{} \frac{x_{1 m}-\mu _m}{\sigma _m} \\ \frac{x_{21}-\mu _1}{\sigma _1} &{} \frac{x_{22}-\mu _2}{\sigma _2} &{} \cdots &{} \frac{x_{n m}-\mu _m}{\sigma _m} \\ \vdots &{} \vdots &{} \ddots &{} \vdots \\ \frac{x_{n 1}-\mu _1}{\sigma _1} &{} \frac{x_{n 2}-\mu _2}{\sigma _2} &{} \ldots &{} \frac{x_{n m}-\mu _m}{\sigma _m} \end{array}\right) . \end{aligned}$$

Here $\mu _j$ denotes the mean and $\sigma _j$ denotes the standard deviation of time series of feature *j* for *n* time points. $c_{jk}$ denotes the correlation between the time series of features *j* and *k*.

8 $$\begin{aligned}{} & {} \mu _j =\frac{1}{n} \sum _{i=1}^n x_{i j}, \end{aligned}$$

9 $$\begin{aligned}{} & {} \sigma _j =\sqrt{\frac{1}{n} \sum _{i=1}^n\left( x_{i j}-\mu _j\right) ^2}, \end{aligned}$$

10 $$\begin{aligned}{} & {} c_{j k} =\frac{\sum _{i=1}^n\left( x_{i j}-\mu _j\right) \left( x_{i k}-\mu _k\right) }{\sigma _j \sigma _k}, \end{aligned}$$

where $$(j\ne k,j,k=1, \ldots ,m)$$, $$c_{jj}=1$$.

We can derive the corresponding correlation magnitude matrix ${\textbf{C}}^*$ from ${\textbf{C}}$ as follows,

11 $$\begin{aligned} {\textbf{C}}^*=\frac{1}{n} {\textbf{X}}_{{\textbf{S}}}^{* T} {\textbf{X}}_{{\textbf{S}}}^*=\left( \begin{array}{cccc} 1 &{} c_{12}^* &{} \cdots &{} c_{1 m}^* \\ c_{21}^* &{} 1 &{} \cdots &{} c_{2 m}^* \\ \vdots &{} \vdots &{} \ddots &{} \vdots \\ c_{m 1}^* &{} c_{m 2}^* &{} \cdots &{} 1 \end{array}\right) , \end{aligned}$$

where ${\textbf{X}}_{{\textbf{S}}}^*$ is defined as,

12 $$\begin{aligned} {\textbf{X}}_{{\textbf{S}}}^*=\left( \begin{array}{cccc} \frac{\left| x_{11}-\mu _1\right| }{\sigma _1} &{} \frac{\left| x_{12}-\mu _2\right| }{\sigma _2} &{} \cdots &{} \frac{\left| x_{1 m}-\mu _m\right| }{\sigma _n} \\ \frac{\left| x_{21}-\mu _1\right| }{\sigma _1} &{} \frac{\left| x_{22}-\mu _2\right| }{\sigma _2} &{} \cdots &{} \frac{\left| x_{2 m}-\mu _m\right| }{\sigma _m} \\ \vdots &{} \vdots &{} \ddots &{} \vdots \\ \frac{\left| x_{n 1}-\mu _1\right| }{\sigma _1} &{} \frac{\left| x_{n 2}-\mu _2\right| }{\sigma _2} &{} \cdots &{} \frac{\left| x_{n m}-\mu _m\right| }{\sigma _m} \end{array}\right) , \end{aligned}$$

and $c_{jk}^*\ge 0,j,k=1, \ldots ,m$. Note that ${\textbf{C}}^*$ is positive semi-definite (PSD)^39^. Eigen-entropy (EE)^36^ is defined as,

13 $$\begin{aligned} \textrm{EE}=-\sum _{i=1}^m \frac{\lambda _i}{m} \log \frac{\lambda _i}{m}, \end{aligned}$$

where $\lambda _i$ is the $i^{th}$ eigenvalue of correlation magnitude matrix ${\textbf{C}}^*$, $\lambda _i\ge 0,i=1, \ldots ,m.$ Let $p_i=\frac{\lambda _i}{m}$ , when we replace the $\frac{\lambda _i}{m}$ term in the EE definition with $p_i$ we get,

14 $$\begin{aligned} \textrm{EE}=-\sum _{i=1}^m p_i \log p_i. \end{aligned}$$

**Figure 2:**
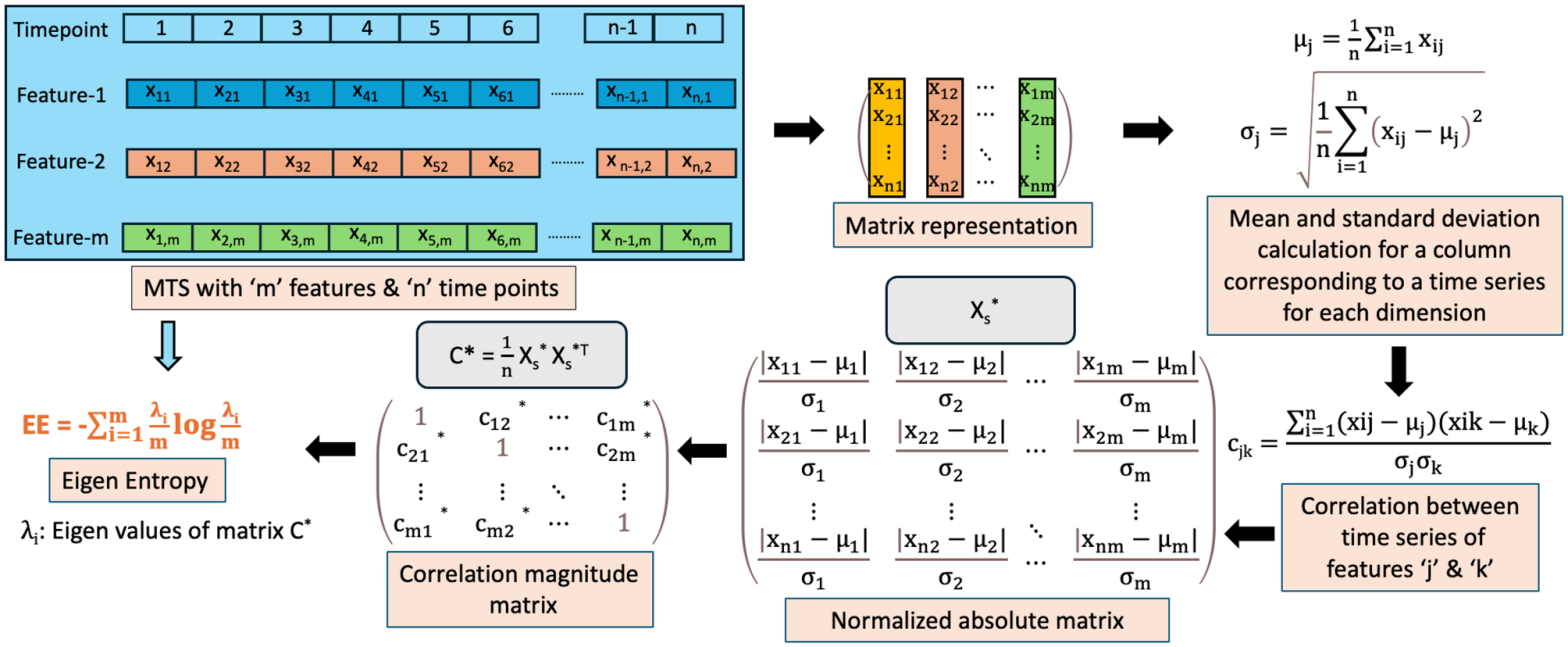
Illustration of Eigen entropy computation.

EE will reach its maximum $p_i=1/m$, or equivalently, when $\lambda _i=1$. Figure 2 below illustrates the process of calculating EE.

It can be proved that as c increases, EE decreases^36^. Considering a time series dataset ${\textbf{X}} \in R^{n \times m}$ with *n* time points, and time series of *m* features as an example, say *EE*(*n*) is the EE of the dataset. When the $(n+1)^{th}$ time point is added to the dataset, if the values corresponding to this new time point increase the variance $\sigma ^2$ of the time series dataset making it more diversified (heterogenous), the magnitude of the correlation (*c*) would decrease, and $EE (n+1)$ would increase, and vice versa.

The Eigen-entropy based Time Series Signatures ($EE-TSS$) is an algorithm proposed to support multivariate time series classification (see [Algorithm 1]). This algorithm needs all the time series in multivariate time series dataset to have equal lengths. In case the involved time series do not have equal lengths, as per the guidelines from A Bier^40^, we pad shorter time series with the last data point in the respective time series to make it equal to the length of the longest time series. This modified time series is called as padded time series.

**Algorithm 1:**
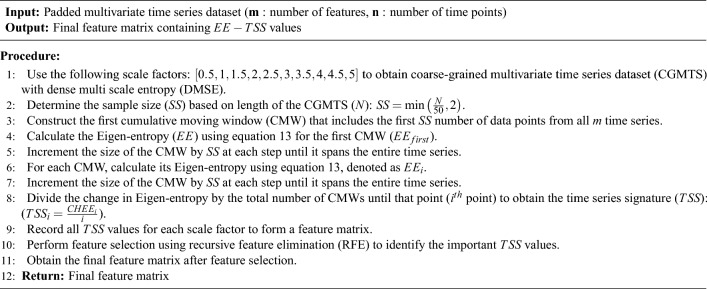
Eigen-entropy based time series signatures ($EE-TSS$)

**Figure 3:**
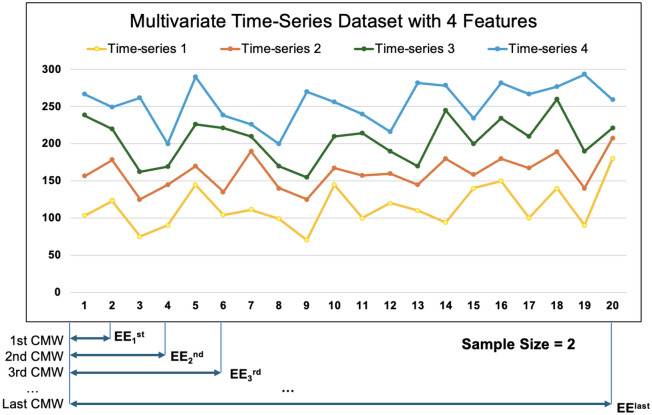
Depiction of CMW for sample size of two.

As presented in Algorithm 1, the feature matrix involves constructing a CMW that spans the entire coarse-grained time series obtained with DMSE (line 1). The size of the CMW is determined by the sample size (*SS*), which is the ratio of the total number of data points in the time series to 50, or 2 if the time series has less than 50 data points (Our experimentation with higher and lower values has shown that 50 is the optimal value since it maintains accuracy without making the algorithm computationally expensive). This process is elaborated with Fig. 3. It displays a randomly generated multivariate time series dataset with four features. To generate the feature matrix at a scale factor of 1 (i.e., for the raw time series), we begin by constructing the first cumulative moving window (CMW) that includes the first 2 data points since total number of data points in this case is less than 50. First 2 data points from all the time series are included to calculate the Eigen-entropy for this CMW, denoted as $EE_{first}$. We then construct the second CMW, which includes the first 4 data points from all-time series, and calculate its Eigen-entropy, denoted as $EE_{second}$. This process is repeated with incrementing the size of the CMW by 2 at each step until it spans the entire time series, with the Eigen-entropy for the final CMW denoted as $EE_{last}$.

Next, the change in Eigen-entropy (*CHEE*) is calculated for each CMW compared to $EE_{first}$ and divide it by the total number of CMWs until that point to obtain the time series signature (*TSS*) (line 7). For example, (*TSS*) for the second CMW is denoted by $TSS_2$ and calculated as $\frac{EE_{second} - EE_{first}}{2}$, while (*TSS*) for the final CMW is denoted as $TSS_{final}$ and calculated as $\frac{EE_{last} - EE_{first}}{\text{ total } \text{ number } \text{ of } \text{ CMWs }}$. All the values for each scale factor are recorded as the multivariate time series signatures.

We perform feature selection using recursive feature elimination (RFE)^41^ to identify the important *TSS* values. The selected features are fed to six classifiers, including support vector machine (SVM), random forest (RF), K nearest neighbors (KNN), logistic regression (LR), Naïve–Bayes (NB), and XGBoost (XGB). These classifiers have been selected due to their extensive application and validation in the domain of supervised machine learning for classification tasks, as corroborated by existing literature^42–46^. The best recall value (Eq. (15)) and the corresponding classifier is compared with the baseline methods, namely Dependent Dynamic Time Warping with K-Nearest Neighbors ($DTWD-KNN$) and Multivariate Multi Scale Permutation Entropy (*MMSPE*) alongwith an appropriate classifier from the aforementioned list.

## Experiments and results

Of particular interest to this research is the disease detection (normal vs. abnormal), which is a binary classification problem. We thus conduct three sets of experiments: the first experiment involves six binary datasets from University of East Anglia (UEA)^37^; the second experiment involves a gait dataset which has two classes namely normal and patients with Parkinson’s disease (PD)^38^; the third experiment consists of an institutional sepsis dataset acquired from the Mayo clinic which has classes normal and sepsis. DTWD is the extension of DTW technique for the multivariate classification, and as DTW with KNN is considered as a golden standard for time series classification^3^, we use DTWD with KNN as one of the baseline classifiers. Another baseline classifier that we selected is based on MMSPE. This choice is motivated by its suitability for multivariate multi scale time series data, similar to our proposed method. Unlike other entropy methods that typically handle univariate or single-scale time series datasets, MMSPE effectively measures correlations and synchronization across multiple channels, which conceptually aligns with our approach. The effectiveness of MMSPE has been demonstrated through graphical comparisons by Morabito^47^ to distinguish between healthy controls and patients with mild cognitive impairment (MCI) and Alzeimer’s disease (AD) using EEG data.

To avoid issues arriving from having non-uniform classifiers, especially for distance-based classifiers such as KNN, the derived $EE-TSS$ values are scaled with min-max scaler. In addition, the labels are encoded as certain classifier algorithms such as SVM, accept only numerical values. The scaled and encoded feature set undergoes recursive feature elimination (RFE)^41^, and the resulting selected features are then provided to various classifiers mentioned earlier (SVM, RF, KNN, LR, NB, and XGB). It is important to note that not every classifier algorithm will perform optimally on all datasets, as each dataset has unique underlying patterns that are best identified by specific types of algorithms. This is known as “no free lunch theorem” in machine learning research. Hence, a diverse set of classifiers is utilized for selection. To ensure a rigorous and equitable comparison, we employed *MMSPE* values across the identical scale factors as $EE-TSS$ (1–5) as features and subjected them to evaluation using the same classifiers under consideration. Following the recommendations delineated in the pertinent literature^47^, we adopted a time lag value of 1 and an embedding dimension of 3 to derive *MMSPE* values for each multivariate time series. Analogous to the $EE-TSS$ procedure, these values are subsequently input into various classifiers. Since recall is the most critical measure for our case, we selected the classifier that produced the best recall, applying the same criteria to our method. Since our research is primarily focused on disease detection, our main focus is on detecting false negatives. Recall, as mentioned in Eq. (15) is a performance metric defined as the ratio of true positive (TP) samples to the total of true positive (TP) and false negative (FN) samples. It ensures judgement of the model performance based on its ability to have minimum FNs. A high number of false negatives indicates that the model may mistakenly identify diseased (positive) individuals as healthy (negative), posing a significant risk to a person’s health. This is why recall is a preferred metric for assessing disease detection models^31,32^. Therefore, drawing on expertise in disease detection and insights from discussions with medical professionals, we have selected recall as the metric to evaluate and compare our algorithm with the $DTWD-KNN$ and *MMSPE* algorithms.

15 $$\begin{aligned} Recall = \frac{TP}{TP+FN}. \end{aligned}$$

While recall serves as a criterion for choosing the best classifier from the set of available classifiers and for assessing classifier performance, it is crucial to ensure that the classifier is not biased. Therefore, we record the area under the ROC (receiver operating characteristics) curve, or simply AUC (area under the ROC curve), which has been traditionally used in medical diagnosis since the 1970s^48,49^. It has been demonstrated as a robust metric for evaluating the predictive capability of learning algorithms. The comparable values of AUC relative to the baseline classifiers allow us to confirm that the developed classifier is not excessively optimized for recall.

To obtain metric values corresponding to each method for each dataset, GridSearchCV with 5-fold stratified cross-validation for hyperparameter tuning of respective classifiers is adopted. The parameters passed to GridSearchCV for each classifier (for classification with our method) are summarized in Table 1. The remaining parameters are set to their default values in Python 3.0 (scikit-learn library version 1.1.3).

**Table 1:** List of parameters for hyperparameter tuning.

Classifiers	Hyperparameters
SVM	kernel: [linear, poly, rbf], C: [1, 10], degree: [2, 3]
RF	n_estimators: [10, 100, 500], criterion: [gini, entropy]
KNN	n_neighbors: [1, 3, 5], weights: [uniform, distance]
NB	Gaussian, var_smoothing: [1e-9, 1e-8, 1e-7, 1e-6, 1e-5]
LR	solvers: [newton-cg, lbfgs, liblinear], penalty: [l2], C: [100, 10, 1.0, 0.1, 0.01]
XGB	n_estimators: [100], learning_rate: [0.3], max_depth: [6], colsample_bytree: [1.0], subsample: [1.0]

### Experiment I: public datasets from the University of East Anglia (UEA)

Six datasets from UEA have equal length time series and do not have any missing values. See Table 2 for details of the dataset. The scale factors employed for preprocessing using DMSE^25^ typically range from 1 to 10. Utilizing scale factors beyond this range may result in the overlap of corresponding entropy values, likely attributable to the insufficient length of the coarse-grained series, and does not necessarily improve diagnostic efficacy. Adhering to the guidelines delineated in the referenced paper, and considering the length of the multivariate series in our case, we selected scale factors between 0.5 and 5 with an increment of 0.5. This selection ensures optimal classification performance without compromising computational efficiency. We generate the feature matrix corresponding to each of the six UEA datasets, as mentioned previously.

**Table 2:** Statistics of 6 experimental UEA datasets.

Datasets	Short-form	Train size	Test size	Number of dimensions	Length of the time series
Face detection	FD	5890	3524	144	62
Finger movements	FM	316	100	28	50
Heartbeat	HB	204	205	61	405
Motor imagery	MI	278	100	64	3000
Self regulation SCP1	SRS1	268	293	6	896
Self regulation SCP2	SRS2	200	180	7	1152

To evaluate the performance of our approach against $DTWD-KNN$ and *MMSPE*, we divided the entire dataset into training and testing sets 30 times, using seeds from 0 to 29 to allow for reproducibility. The ratio for the train-test split was 80 : 20. Additionally, we ensured the samples were stratified during the splitting process, following the method of AP Ruiz^3^. For classification with $DTWD-KNN$, 1 nearest neighbor is used as done by AP Ruiz^3^. We consider types of weights as uniform, and distance to cross-validate and hyper tune parameters with GridSearchCV. We then compute the recall and AUC metrics for the classifier. For *MMSPE*, the computed entropy values are provided to the aforementioned six classifiers to ensure a fair comparison, and recall values are calculated for all six classifiers. The classifier with the highest recall value is then selected, and the AUC for this classifier is computed. Finally, the mean AUC and recall values for both baseline methods and $EE-TSS$ are calculated and presented in Tables 3 and 4, respectively. The optimal classifiers for the six datasets under examination are enumerated as follows: Hertbeat-NB, Face Detection-RF, Finger Movements-KNN, MotorImagery-SVM, Self Regulation SCP1-LR, Self Regulation SCP2-XGB for MMPSE-based analysis, and Hertbeat-NB, Face Detection-LR, Finger Movements-NB, MotorImagery-NB, Self Regulation SCP1-NB, Self Regulation SCP2-KNN for $EE-TSS$-based analysis. UEA datasets examined in this study can be accessed on the embedded link here: UEA. [We utilized the code from Donets, N. (2013)-PyEntropy Github repository, and Alberto, A. (2023)- Multivariate-multiscale-permutation-entropy Github repository for implementation of MMSPE.]

**Table 3:** AUC metrics for all eight datasets under review (’*’: NaN values).

Dataset	DTWD-KNN	MMSPE	EE-TSS
Heartbeat	0.57	0.66	0.67
Face detection	0.53	0.00*	0.50
Finger movements	0.54	0.50	0.52
Motor imagery	0.51	0.48	0.49
Self regulation SCP1	0.81	0.59	0.79
Self regulation SCP2	0.55	0.52	0.59
Gait	0.52	0.56	0.59
Sepsis Pig	0.69	0.66	0.30

**Table 4:** Recall metrics for all eight datasets under review.

Dataset	DTWD-KNN (%)	MMSPE (%)	EE-TSS (%)
Heartbeat	30	28	64
Face detection	49	0	53
Finger movements	56	54	64
Motor imagery	52	53	60
Self regulation SCP1	80	54	82
Self regulation SCP2	62	54	60
Gait	52	91	77
Sepsis Pig	69	95	96

### Experiment II: gait

The data utilized in this research is obtained from ‘The PhysioBank^38^’. This repository comprises gait measurements from 93 individuals diagnosed with idiopathic parkinson’s disease (PD), and 73 healthy controls. The dataset consists of the vertical ground reaction force data of participants while walking at their typical pace for about 2 minutes on flat terrain. Each foot is equipped with 8 sensors that capture force (measured in Newtons) over time. The readings from these 16 sensors have been digitized and sampled at a rate of 100 times per second. Additionally, the dataset includes two signals representing the combined output of the 8 sensors for each foot. Some patients have completed only one walking trial, whereas others have completed multiple trials. To ensure consistency, only the initial walking trial for all individuals is included in the study. Seventy two unique individuals out of 73 are selected based on our analysis. Since the values of features remain relatively stable across ten consecutive time points, their average values are computed. In this dataset, the time series lengths vary among subjects, with each series containing around an average of 2000 time points prior to preprocessing. Bier^40^ proposes three strategies to make the multivariate time series of equal length: padding, truncation, and forecasting with auto regressive integrated moving average (ARIMA) and concludes that padding (with a constant value) is the best strategy. Consequently, we employ padding to standardize the length of the time series, aligning the shorter sequences with the longest one. Following the preprocessing of the dataset, the subsequent methodology employed to derive the metric values aligns with the procedures utilized for UEA datasets. Table 3 delineates the mean AUC, and Table 4 delineates the mean recall obtained using $EE-TSS$ algorithm together with two baseline algorithms. The most efficacious classifier for this dataset is LR when analyzed using the *MMSPE* methodology, whereas the SVM demonstrates superior performance under the $EE-TSS$ analytical framework. [The gait dataset examined in this study can be accessed on the embedded link here: Gait.]

### Experiment III: institutional sepsis dataset

Acute Kidney Injury (AKI) begins without clinical symptoms or signs and occurs in up to $20\%$ of hospital admissions^50–53^ resulting in  2 million patients per year^54^ and extra inpatient costs. AKI is generally not recognized early since the markers used to diagnose AKI (i.e., serum creatinine) change only after global dysfunction has significantly evolved. The physiology has not been extensively explored or corresponding sensors have not been developed to monitor AKI. To fill this gap, the Mayo Clinic has created the device capable of automated measurement of certain biomarkers. The use of the device in our swine AKI experiments demonstrate that under continuous monitoring, these biomarkers change rapidly under AKI prior to the change in traditional markers (e.g., blood creatinine) and hence have the potential for early AKI or sepsis detection^55^. The dataset consists of two classes. The first class corresponds to control category, and the later one corresponds to sepsis category. Swine under the control category have normal physiological behavior whereas swine under sepsis category are infused with E-Coli to artificially generate septic shock simulating sepsis condition. There is total 12 swine corresponding to the control category and 24 swine corresponding to the sepsis category. We are using multivariate time series corresponding to the biomarkers. The readings are taken at an interval of 20-min. The data has been collected over the duration of few hours. For this dataset, the lengths of the time series are different corresponding to all the swine due to different life span of the swine involved in the experiments. We utilize padding^40^ to equalize the time series lengths, extending the shorter sequences to match the longest one. Following the padding process, the subsequent methodology mirrors that utilized for the UEA and gait datasets. Table 3 presents the mean AUC, and Table 4 presents the mean recall achieved using $EE-TSS$ algorithm in conjunction with two baseline algorithms. The LR classifier exhibits the highest efficacy for this dataset when evaluated using both the *MMSPE*, and $EE-TSS$ algorithms. [The institutional sepsis data that supports the findings of this study is available from Mayo Clinic in Arizona but restrictions apply to the availability of this dataset as the dedicated publication is being processed, which was used under license for the current study, and so is not publicly available. Dataset is, however, available from the authors upon reasonable request and with permission of Mayo Clinic in Arizona.]

### Ethics declarations-approval for animal experiments

All experiments are conducted with the approval of the Institutional Animal Care and Use Committee (IACUC). All animals are treated in accordance with the Guide for the Care and Use of Laboratory Animals. The research is conducted in an OLAW-assured, AAALAC-accredited, USDA-registered facility. We certify that ARRIVE guidance has been followed.

**Figure 4:**
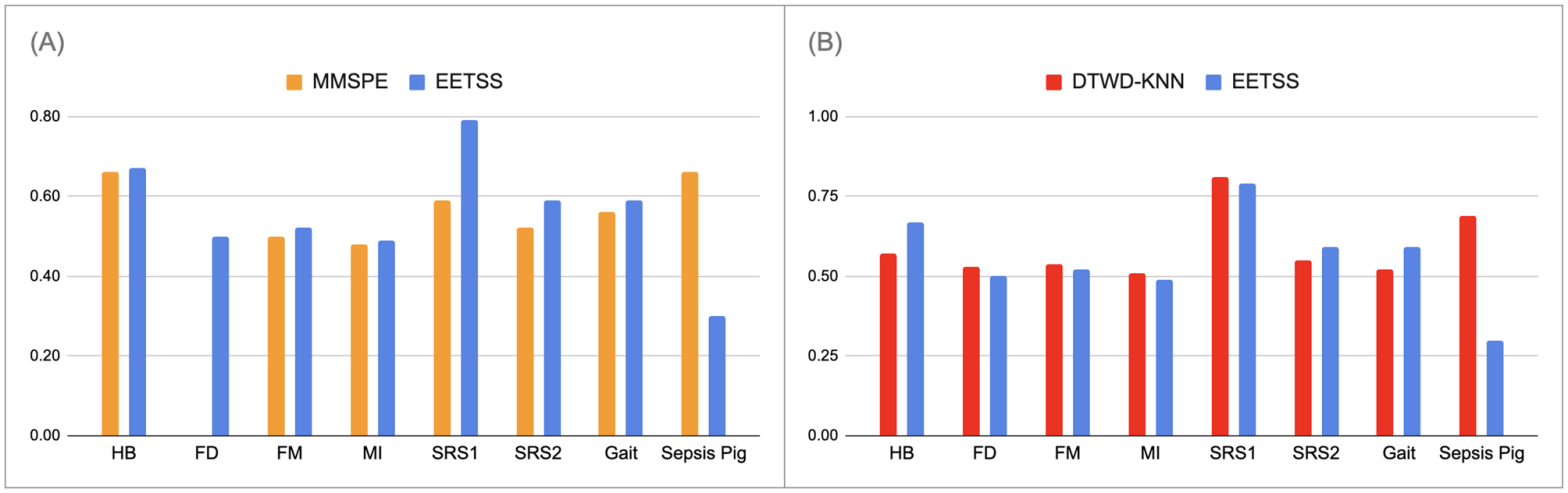
AUC metrics for: (**A**) *MMSPE* and $EE-TSS$ algorithms, (**B**) $EE-TSS$ and $DTWD-KNN$ algorithms for eight datasets under consideration.

**Figure 5:**
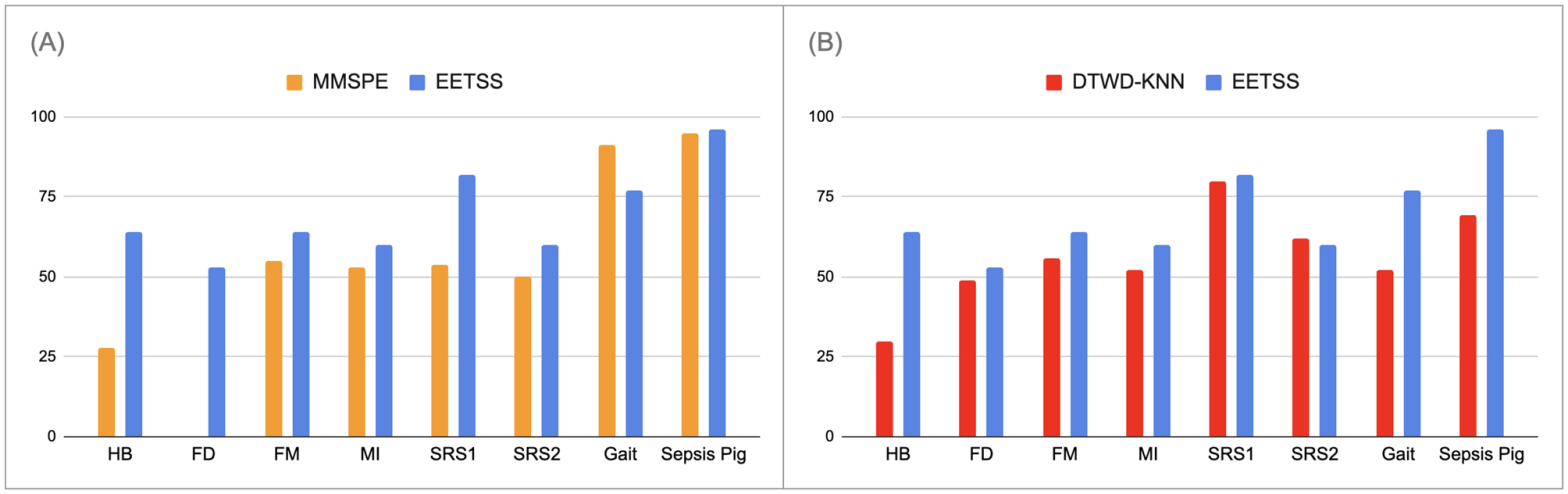
Recall metrics for: (**A**) *MMSPE* and $EE-TSS$ algorithms, (**B**) $EE-TSS$ and $DTWD-KNN$ algorithms for eight datasets under consideration.

The AUC and recall metrics for all eight datasets are illustrated in the following figures (Figs. 4, 5) respectively. These visualizations provide a clearer comparison of the performance of three different algorithms ($EE-TSS$, and two baseline algorithms: *MMSPE* and $DTWD-KNN$). By examining these figures, we can gain a deeper understanding of how these algorithms perform relative to one another. The $EE-TSS$ algorithm outperforms $DTWD-KNN$ and *MMSPE* in recall for 7 out of 8 datasets. This ensures more effective identification of relevant instances, crucial in applications like disease detection, where missing true positives is costly. The AUC values for our algorithm surpass those of *MMSPE* in 7 out of 8 datasets and exceed those of $DTWD-KNN$ in the HB, SRS2, and Gait datasets. Further analysis revealed that the AUC values for FM and MI, show no statistical difference between the $DTWD-KNN$ and $EE-TSS$ methods, as confirmed by Welch’s unpaired t-test^56^ (p > 0.05). Thus, we conclude that our algorithm performs better or is equivalent to $DTWD-KNN$ in 5 out of 8 datasets. This demonstrates the overall outperformance of our classifier in terms of AUC compared to the baseline algorithms. These strengths highlight $EE-TSS$ as a well-rounded and innovative approach, providing significant practical benefits by enhancing the reliability and effectiveness of systems in real-world scenarios where high recall is critical. Therefore, $EE-TSS$ proves to be a valuable advancement worth adopting over existing methods.

## Discussion

The $EE-TSS$ algorithm demonstrates higher recall compared to $DTWD-KNN$ and *MMSPE* on most datasets while preserving competitive AUC. Additionally, our proposed algorithm’s feature generation is achieved through a relatively simple and thus understandable method. This can provide clinicians with the necessary confidence for using it in clinical disease prediction tasks. Furthermore, the $EE-TSS$ algorithm requires minimal data for training, as it performs well with only a few subjects and a small number of time points. This makes the algorithm well-suited for clinical trial datasets, which often have fewer subjects and shorter time series.

It is noteworthy that our methodology effectively classifies UEA datasets, including those not explicitly associated with disease detection. This efficacy arises from the time series signatures, which encapsulate the extent of asynchronization among various time series of multiple variables within the multivariate dataset, exhibiting markedly distinct values for the diverse classes involved in the classification process.

Our algorithm fundamentally hinges on deriving entropy values from the correlations observed in the concurrent movement of time series features, emphasizing intra-subject dependencies. We hypothesize that the integration of features accounting for inter-subject dependencies could substantially augment performance. Additionally, our research, which is centered on disease detection, addresses a binary classification problem wherein entropy estimation quantifies correlations between features that distinguish healthy individuals from those afflicted with disease. Although this binary approach is straightforward, extending it to multiclass classification, such as disease subtyping, might necessitate additional strategies, such as the one-vs-one or one-vs-rest methodologies employed in SVM-like algorithms. These strategies could assist in managing overlapping correlation patterns. Thus, extending this to multiclass classification represents a promising research direction. Furthermore, we employ fixed scaling factors for all multivariate time series, however, investigating adaptive scaling to determine scale factors for different series could uncover critical information and enhance classification performance, presenting another crucial avenue for future research.

## Conclusion

In summary, this study elucidates the limitations inherent in contemporary multivariate time series classification algorithms which do not consider the interdependence among time series features and the inadequacies of entropy-based algorithms in the domain of multivariate time series classification. To mitigate these issues, we employ Eigen-entropy-based time series signatures to quantify feature correlations, while also addressing the temporal characteristics of the time series data through the incorporation of a cumulative moving window. To further accommodate the dynamic nature of time series data, we integrate dense multi-scale entropy. Additionally, we conduct feature selection via recursive feature elimination and apply conventional machine learning algorithms for classification. To validate the efficacy of our methodology, we assess its performance on six UEA datasets, a gait dataset, and an institutional sepsis dataset. The algorithm exhibits robust classification performance across all evaluated datasets, achieving superior recall, and commendable AUC, thereby underscoring its effectiveness in disease detection and binary classification tasks. To further improve its performance, we could incorporate features that capture inter-subject dependencies and implement adaptive scaling. Additionally, this methodology could be extended to multiclass classification, enabling tasks such as disease subtyping through the use of additional strategies.

## Data Availability

The UEA and public gait datasets analysed during the current study are available in the University of East Anglia (UEA), and “PhysioBank, PhysioToolkit, and PhysioNet: Components of a new research resource for complex physiologic signals” repositories, and are available at UEA, Gait respectively. The institutional sepsis data that supports the findings of this study are available from Mayo Clinic in Arizona but restrictions apply to the availability of these data as the dedicated publication is being processed, which were used under license for the current study, and so are not publicly available. Data are however available from the authors upon reasonable request and with permission of Mayo Clinic in Arizona.
